# Direct and Functional Hazard Exposure of Urban Healthcare Systems Under Estimated Tsunami Inundation Zones: A Geographic Information System (GIS)-Based Cross-Boundary Analysis in Osaka City, Japan

**DOI:** 10.7759/cureus.108053

**Published:** 2026-04-30

**Authors:** Kentaro Tanaka, Ryo Horiike, Itatani Tomoya, Hisao Nakai

**Affiliations:** 1 Nursing, Osaka Metropolitan University, Osaka, JPN; 2 Nursing, Nara Medical University, Nara, JPN; 3 School of Nursing, Faculty of Medicine, University of Miyazaki, Miyazaki, JPN; 4 Faculty of Nursing, University of Kochi, Kochi, JPN

**Keywords:** disaster preparedness, geographic information systems, healthcare system, medical institutions, tsunami, vulnerability

## Abstract

Background

Tsunami disasters pose significant challenges to urban healthcare systems, particularly in densely populated coastal metropolitan areas. Although previous studies have examined hazard exposure and evacuation dynamics, limited attention has been paid to how healthcare system vulnerability arises from the combined effects of hazard exposure and population redistribution. This study examined the spatial relationships among estimated tsunami inundation zones, population distribution, and healthcare institutions in Osaka City, Japan, to identify structural vulnerabilities in urban healthcare systems.

Methods

A cross-sectional geographic information system (GIS)-based spatial analysis was conducted using publicly available secondary data from official sources, including the Hazard Map Portal Site (MLIT) and national census data (e-Stat). Spatial relationships among estimated tsunami inundation zones, population distribution, evacuation-related facilities, and medical institutions were examined. Spatial overlay analysis identified medical institutions located within inundation zones, and a nearest-neighbor approach was used to estimate changes in the population served by each institution under tsunami conditions.

Results

The analysis suggests a dual structure of healthcare system vulnerability. First, several healthcare institutions in Nishinari Ward were located within estimated tsunami inundation zones, indicating direct physical exposure and potential functional disruption. Second, medical institutions located outside inundation zones experienced substantial increases in the surrounding population under tsunami conditions, with some institutions showing up to a 5.5-fold increase compared to baseline levels. This reflects indirect functional exposure due to concentrated healthcare demand.

Conclusion

These findings suggest that disaster vulnerability in urban healthcare systems can be characterized by a dual structure consisting of direct physical exposure and indirect functional exposure. In large coastal metropolitan areas, effective disaster preparedness should consider not only static hazard exposure but also dynamic processes such as population redistribution and cross-boundary healthcare demand. Integrating these perspectives is important for developing more resilient healthcare planning strategies.

## Introduction

Tsunami disasters pose substantial risks to coastal urban areas. In densely populated cities, these impacts can be particularly severe because evacuation systems are often complex and constrained [1-3]. High population density, limited evacuation routes, and the spatial concentration of residents may amplify threats to health and safety during tsunami events.

In Japan, tsunami inundation hazard maps are widely disseminated and publicly available to support disaster preparedness and evacuation planning at the municipal level [4]. These maps indicate areas expected to be inundated based on modeled tsunami scenarios and are intended to encourage timely evacuation. From a risk communication perspective, hazard information functions as an environmental and social cue that shapes residents’ risk perception and influences protective decision-making [5]. Empirical studies on disaster risk perception, including those on flood preparedness, indicate that both cognitive and affective responses play important roles in shaping protective actions [6]. However, the effectiveness of such decision-making also depends on the spatial configuration of hazards, population distribution, and evacuation infrastructure. In large metropolitan areas, tsunami inundation zones often overlap with residential areas and critical facilities, raising concerns about the spatial alignment of evacuation-related facilities with hazard exposure.

To address these spatial challenges, previous studies have applied geographic information system (GIS)-based approaches and evacuation modeling methods to assess tsunami risk, population exposure, and evacuation feasibility. These approaches include analyses based on pedestrian travel time and least-cost path assumptions [7-9]. By visualizing spatial relationships among hazards, population distribution, and evacuation routes, these methods have contributed to evidence-based disaster preparedness planning. However, many studies focus on a single administrative unit or evaluate evacuation systems within fixed administrative boundaries.

In practice, evacuation behavior during large-scale disasters is not necessarily confined to administrative boundaries, particularly when hazard zones extend across adjacent jurisdictions. For example, spatial analyses in Alameda, California, have shown that residents in high-risk areas may evacuate to neighboring jurisdictions, highlighting the importance of interjurisdictional coordination in tsunami planning [10]. Analyses limited to administrative units may therefore overlook important cross-boundary evacuation dynamics in metropolitan areas.

In addition to evacuation systems, healthcare institutions play a critical role in disaster response by providing medical care to injured individuals, patients with chronic conditions, and other vulnerable populations. However, limited research has examined how the spatial relationship between tsunami hazards and healthcare institutions affects the distribution of healthcare demand, particularly when some institutions become non-functional during disasters.

Therefore, this study examined the spatial overlap and distribution of estimated tsunami inundation zones, residential population, evacuation-related facilities, and healthcare institutions in two adjacent urban wards of Osaka City, Japan, the second-largest municipality in Japan by population [11]. Using a cross-sectional GIS-based analysis of publicly available data, this study aimed to (1) identify healthcare institutions located within estimated tsunami inundation zones (direct physical exposure) and (2) quantify changes in the population served by non-exposed institutions under tsunami conditions using a nearest-neighbor allocation model (indirect functional exposure). These analyses provide a basis for understanding cross-boundary redistribution of healthcare demand and its implications for disaster preparedness in urban coastal settings.

## Materials and methods

Study design and study area

A cross-sectional spatial analysis was conducted using secondary data to examine the spatial relationships among estimated tsunami inundation zones, population distribution, evacuation-related facilities, and medical institutions in Osaka City, Japan.

The study area comprised two adjacent administrative wards with contrasting tsunami hazard characteristics: Nishinari Ward and Abeno Ward. Nishinari Ward includes extensive estimated tsunami inundation zones, whereas most areas of Abeno Ward are located outside these zones. Although geographically adjacent, these wards also differ in underlying socioeconomic and health-related conditions, providing a contrasting urban context for examining healthcare system vulnerability.

Selecting these two wards enabled comparison of spatial patterns of tsunami inundation, residential population distribution, evacuation-related facilities, and medical institutions under different hazard conditions within the same metropolitan area. Furthermore, focusing on neighboring administrative units with different hazard exposure conditions allowed examination of cross-boundary redistribution of population and healthcare demand during tsunami events. All analyses were conducted using mesh-level spatial data, with ward-level interpretation applied for contextual purposes.

Data sources

Estimated Tsunami Inundation Zones

Spatial data were obtained from the Hazard Map Portal Site operated by the Ministry of Land, Infrastructure, Transport and Tourism (MLIT), Japan, which provides official national hazard datasets. The data available as of May 2025 were used (accessed April 1, 2026) [4]. These datasets were used as polygon data representing areas expected to be inundated under modeled tsunami scenarios.

Healthcare institutions located within the estimated tsunami inundation zones were classified as exposed based on hazard map data. This classification did not consider specific inundation depth thresholds, and any facility located within the inundation polygon was treated as exposed.

Population Data

Population distribution was analyzed using 250-m mesh data from the 2020 Population Census, obtained from e-Stat (accessed April 1, 2026) [12]. These data were used to assess spatial overlap between residential population and estimated tsunami inundation zones. The 2020 Census represents the most recent available 250-m mesh dataset in Japan; therefore, no additional temporal adjustment was applied. As this study used publicly available standardized datasets from official sources, no additional data imputation or preprocessing procedures were performed.

Evacuation-Related Facilities

Data were obtained from publicly available datasets provided by the Geospatial Information Authority of Japan (GSI) (accessed April 1, 2026) [13]. Facilities were classified into two groups: (1) those designated only as Designated Emergency Evacuation Sites, and (2) those designated as both Designated Emergency Evacuation Sites and Designated Evacuation Shelters.

Medical Institutions

Location data were obtained from the 2020 National Land Numerical Information (NLNI) dataset provided by the MLIT (accessed April 1, 2026) [14]. This dataset includes point data representing hospital-level medical institutions. The most recent available data were used.

Medical institutions included in this study were general hospitals in Japan identified from publicly available datasets. Clinics and smaller outpatient facilities were excluded to ensure consistency in facility classification.

All facility data were provided as point data and analyzed separately for Nishinari and Abeno wards. Administrative boundary data were obtained from the NLNI [14], and population mesh data were retrieved from e-Stat [12]. Background maps were created using GSI Standard Map Tiles (accessed April 1, 2026) [15].

Data Usage and Licensing

All datasets are publicly available and permitted to be used with appropriate attribution. Data were used in accordance with these terms.

GIS analysis

All GIS analyses were conducted using QGIS (version 3.40; QGIS Development Team) with standard spatial analysis procedures. Spatial datasets were projected to the Japan Geodetic Datum 2011 (JGD2011) plane rectangular coordinate system (Zone II).

Spatial overlay procedures

Population Analysis

Spatial overlay was conducted between 250-m population mesh polygons and estimated tsunami inundation zone polygons. Meshes intersecting inundation zones were classified as overlapping. For each ward, the number and proportion of overlapping meshes and corresponding population totals were calculated.

Evacuation-Related Facilities

Facility point data were overlaid with inundation polygons. Facilities located within inundation zones were classified as exposed.

Medical Institutions and Definition of Hazard Exposure

Medical institution point data were overlaid with inundation polygons. Hazard exposure was defined as a medical institution located within an estimated tsunami inundation zone. Such institutions were classified as exposed, while those outside were classified as non-exposed.

Estimation of Population Served by Medical Institutions

Population mesh centroids were assigned to medical institutions using a nearest-neighbor allocation approach based on Euclidean distance (straight-line distance). Distance was calculated between the centroid of each 250-m population mesh and each medical institution. Administrative ward boundaries were not enforced, allowing cross-boundary allocation to the nearest facility. In cases of equal distance, one facility was assigned arbitrarily, as such cases were rare and did not materially affect the results. Under normal conditions, each mesh centroid was linked to the closest medical institution, and the population served by each institution was calculated. In contrast, under tsunami conditions, medical institutions located within inundation zones were assumed to be non-functional and excluded; population mesh centroids were then reassigned to the nearest non-exposed medical institution, and the resulting population was calculated as the potential demand during tsunami events.

This simplified approach was adopted to capture relative spatial patterns of accessibility, and its potential impact is addressed in the limitations. All analytical procedures were conducted using standard GIS operations. The datasets used in this study are publicly available, and the analytical workflow can be conceptually reproduced based on the procedures described above.

The analysis was conducted at the mesh level (250-m population mesh), and population allocation was estimated at the level of individual medical institutions. Ward-level interpretation was used only for descriptive and contextual purposes. Intra-ward heterogeneity was inherently captured through mesh-level analysis rather than aggregated statistics. Cross-boundary allocation was permitted; therefore, edge effects near ward boundaries were inherently incorporated into the analysis.

Ethical considerations

This study used publicly available secondary data and did not involve human subjects. No personal or identifiable information was included. Therefore, ethical review and informed consent were not required.

## Results

Study area and estimated tsunami inundation zones

**Figure 1:**
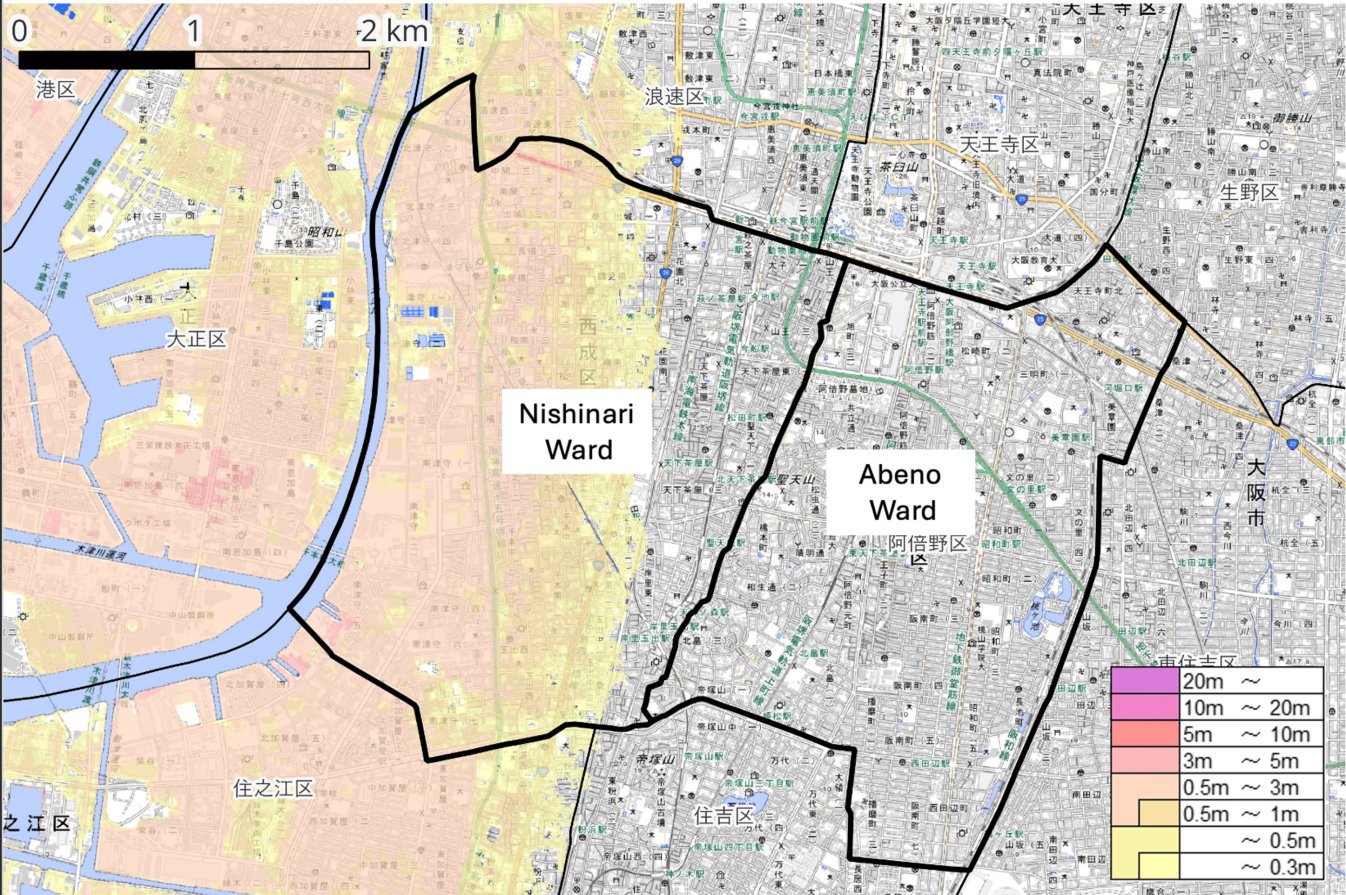
Study area and projected tsunami inundation zones in Nishinari and Abeno Wards, Osaka City, Japan Thick black lines indicate administrative boundaries. Areas shaded from yellow to light red represent the estimated tsunami inundation zones. The color bar indicates the estimated tsunami inundation depth (m) based on hazard map data. The color gradient represents the range of inundation depths, with darker colors indicating greater inundation depth. Administrative boundary data were obtained from the National Land Numerical Information (NLNI) [14]. Data on the estimated tsunami inundation zones were obtained from the Hazard Map Portal Site operated by the Ministry of Land, Infrastructure, Transport and Tourism (MLIT), Japan [4]. The base map data were derived from the GSI Standard Map Tiles provided by the Geospatial Information Authority of Japan (GSI) [15]. This figure was created by the authors using publicly available datasets and QGIS (version 3.40; QGIS Development Team).

This study examined two adjacent administrative wards in southern Osaka City: Nishinari Ward and Abeno Ward. As shown in Figure 1, estimated tsunami inundation zones were widely distributed in the western part of Nishinari Ward, whereas most areas of Abeno Ward were located outside these zones. In Nishinari Ward, inundation-risk areas formed a spatially continuous zone extending along the coastal side of the ward.

Population distribution within estimated tsunami inundation zones

**Figure 2:**
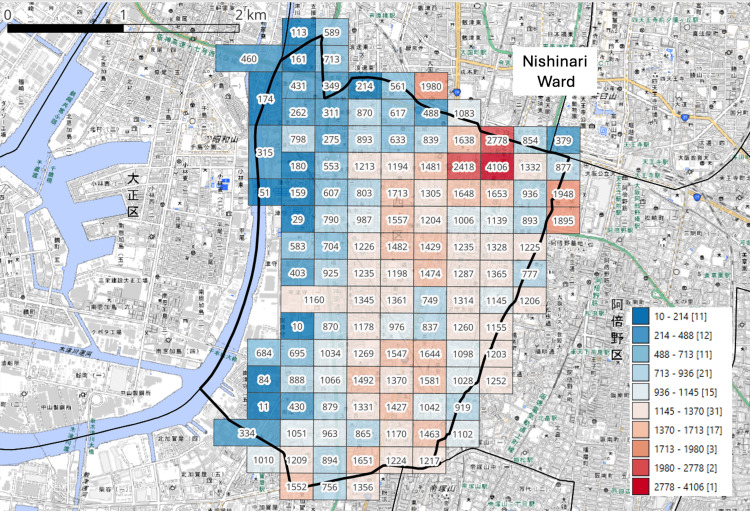
Population Distribution at the 250-m Mesh Level in Nishinari Ward, Osaka City, Japan Thick black lines indicate administrative boundaries. The grid of square cells represents the population at the 250-m mesh level, and the numbers indicate the population within each mesh. The values shown to the right of the color bar indicate the population per mesh, and the numbers in brackets indicate the number of meshes within each category. Administrative boundary data were obtained from the National Land Numerical Information (NLNI) [14]. Population data (2020 Population Census, 250-m mesh) were obtained from e-Stat [12]. The base map data were derived from the GSI Standard Map Tiles provided by the Geospatial Information Authority of Japan (GSI) [15]. This figure was created by the authors using publicly available datasets and QGIS (version 3.40; QGIS Development Team).

Analysis of 250-m mesh population data revealed a substantial concentration of residents within estimated tsunami inundation zones in Nishinari Ward (Figure 2). Of the 124 population mesh cells, 90 (72.6%) overlapped with inundation zones. The population within these areas was estimated at 79,995 residents, representing approximately 63.1% of the ward’s total population.

In contrast, 34 mesh cells (27.4%) were located outside the inundation zones, with a population of 46,693 residents. These non-inundated areas were primarily located in the eastern part of Nishinari Ward, near the boundary with Abeno Ward.

Cross-boundary evacuation structure and evacuation facility functions

**Figure 3:**
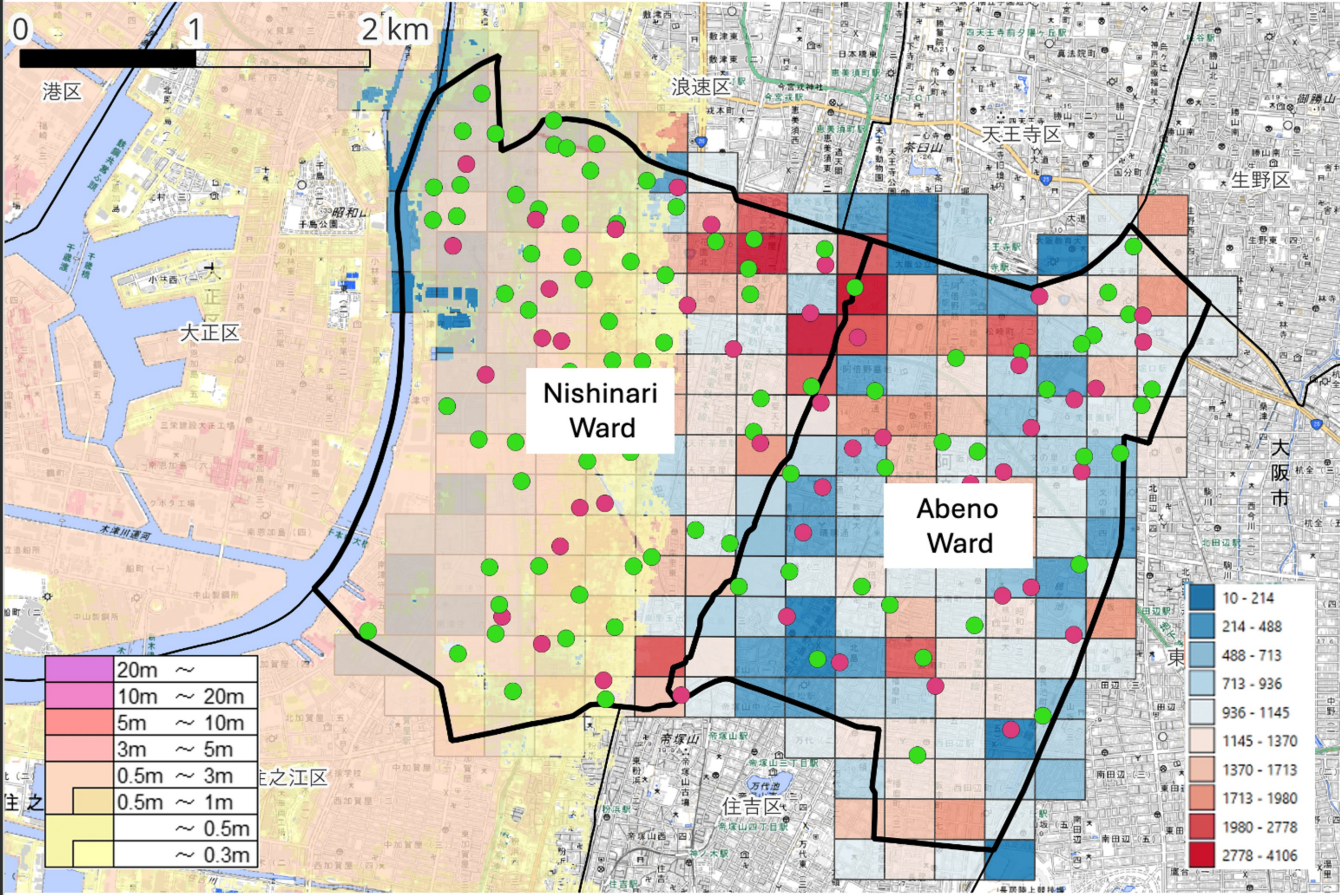
Distribution of Designated Evacuation Shelters in Relation to Population Distribution and Estimated Tsunami Inundation Zones in Nishinari and Abeno Wards, Osaka City, Japan Thick black lines indicate administrative boundaries. Areas shaded from yellow to light red represent the estimated tsunami inundation zones. The color bar on the left indicates the estimated tsunami inundation depth (m) based on hazard map data; the color gradient represents the range of inundation depths, with darker colors indicating greater inundation depth. The grid of square cells represents the population at the 250-m mesh level. The values shown in the color bar on the right indicate the population in each mesh, with darker colors representing higher population. Green circles indicate Designated Emergency Evacuation Sites, and magenta circles indicate facilities designated as both Designated Emergency Evacuation Sites and Designated Evacuation Shelters. Administrative boundary data were obtained from the National Land Numerical Information (NLNI) [14]. Data on the estimated tsunami inundation zones were obtained from the Hazard Map Portal Site operated by the Ministry of Land, Infrastructure, Transport and Tourism (MLIT), Japan [4]. Population data (2020 Population Census, 250-m mesh) were obtained from e-Stat [12]. Data on Designated Emergency Evacuation Sites and facilities designated as both Designated Emergency Evacuation Sites and Designated Evacuation Shelters were obtained from the database provided by the Geospatial Information Authority of Japan (GSI) [13]. The base map data were derived from the GSI Standard Map Tiles [15]. This figure was created by the authors using publicly available datasets and QGIS (version 3.40; QGIS Development Team).

Figure 3 shows the spatial overlay of inundation zones, population distribution, administrative boundaries, and evacuation-related facilities. In Abeno Ward, 53 evacuation-related facilities were identified, including 28 designated only as Designated Emergency Evacuation Sites and 25 designated as both Designated Emergency Evacuation Sites and Designated Evacuation Shelters. In Nishinari Ward, 84 facilities were identified, including 61 designated only as Designated Emergency Evacuation Sites and 23 designated as both.

The spatial distribution of facilities differed between the two wards. In Nishinari Ward, many facilities were located within or near inundation zones, whereas in Abeno Ward, most were located outside these zones. This configuration suggests evacuation flows from inundation-risk areas in Nishinari Ward toward inland areas, including across the administrative boundary into Abeno Ward.

Population distribution surrounding medical institutions inside and outside estimated tsunami inundation zones

**Figure 4:**
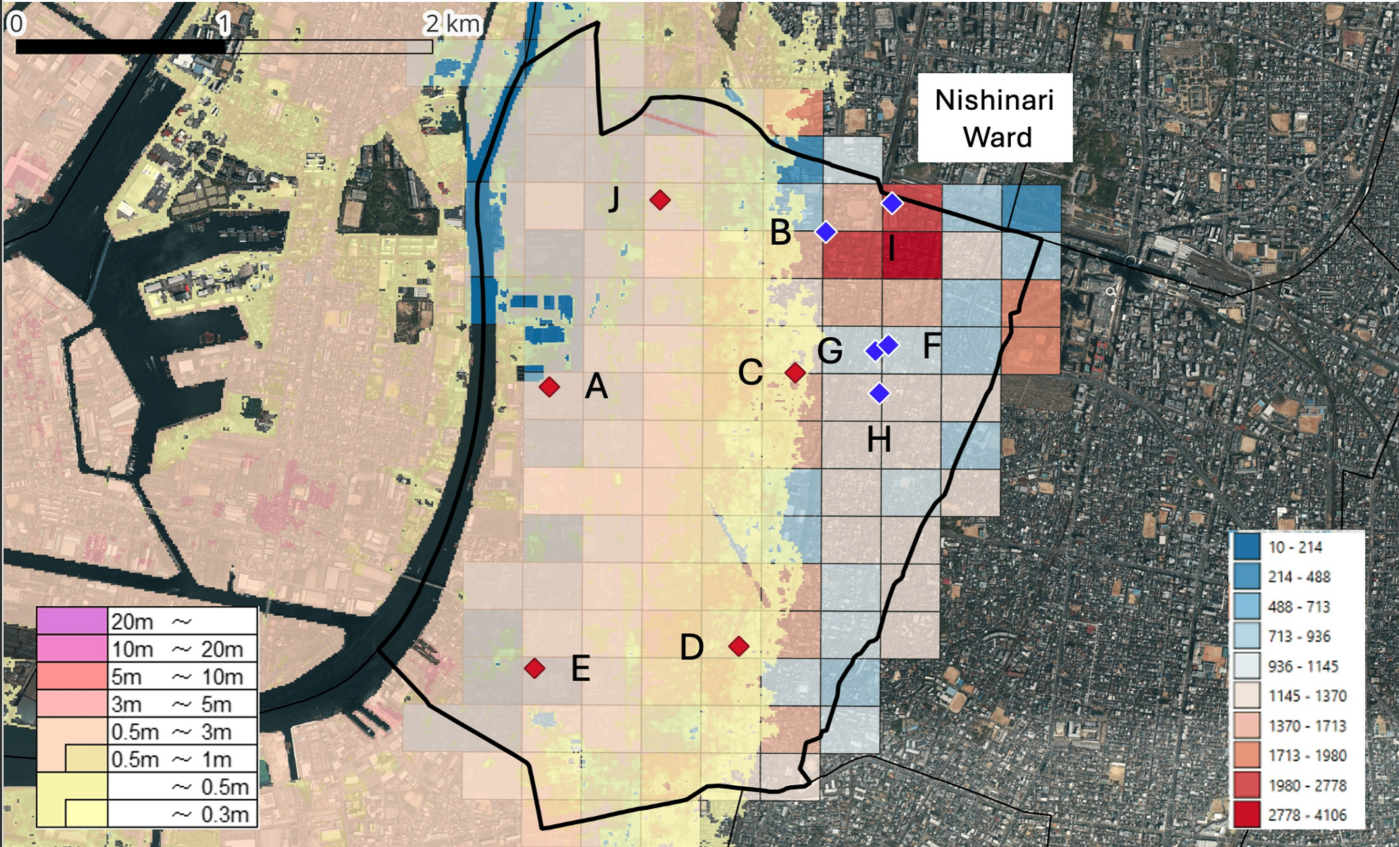
Locations of Medical Institutions and Their Exposure to Estimated Tsunami Inundation Zones in Nishinari Ward, Osaka City, Japan Thick black lines indicate administrative boundaries. Areas shaded from yellow to light red represent the estimated tsunami inundation zones. The color bar on the left indicates the estimated tsunami inundation depth (m) based on hazard map data; the color gradient represents the range of inundation depths, with darker colors indicating greater inundation depth. The grid of square cells represents the population at the 250-m mesh level. The values shown in the color bar on the right indicate the population in each mesh, with darker colors representing higher population. Labels A–J indicate the locations of medical institutions. Red diamonds indicate medical institutions located within the estimated tsunami inundation zones, while blue diamonds indicate those located outside the inundation zones. Administrative boundary and medical institution data were obtained from the National Land Numerical Information (NLNI) [14]. Data on the estimated tsunami inundation zones were obtained from the Hazard Map Portal Site operated by the Ministry of Land, Infrastructure, Transport and Tourism (MLIT), Japan [4]. Population data (2020 Population Census, 250-m mesh) were obtained from e-Stat [12]. The base map data were derived from the GSI Seamless Photo (latest nationwide imagery) provided by the Geospatial Information Authority of Japan (GSI) [15]. This figure was created by the authors using publicly available datasets and QGIS (version 3.40; QGIS Development Team).

Medical institutions were classified based on their spatial relationship with estimated tsunami inundation zones. For each institution (Hospitals A-J), the surrounding population was estimated under both normal and tsunami conditions. Hospitals A, C, D, E, and J were located within inundation zones, indicating potential direct physical exposure and functional disruption (Figure 4).

In contrast, medical institutions located outside inundation zones (Hospitals B, F, G, H, and I) were not directly exposed but showed substantial increases in the surrounding population under tsunami conditions (Table 1). For example, the estimated surrounding population of Hospital H increased from approximately 13,297 under normal conditions to approximately 72,946 under tsunami conditions, representing an approximately 5.5-fold increase. Although the magnitude varied, all non-exposed institutions showed increased surrounding population under tsunami conditions.

**Table 1:** Comparison of nearby population around medical institutions under baseline and tsunami conditions Values represent the estimated number of residents within the defined catchment area of each medical institution. NA indicates not applicable because the institution is located within estimated tsunami inundation zones.

Hospital ID	Nearby population (baseline)	Nearby population (tsunami, non-inundated institutions)	Ratio (tsunami/baseline)
A	9,217	Inundated (NA)	–
B	8,844	27,750	3.1
C	14,459	Inundated (NA)	–
D	31,242	Inundated (NA)	–
E	14,420	Inundated (NA)	–
F	8,464	8,464	1
G	2,654	6,119	2.3
H	13,297	72,946	5.5
I	11,409	11,409	1
J	12,682	Inundated (NA)	–

## Discussion

This GIS-based spatial analysis of two adjacent administrative wards in Osaka City identified a dual structure of hazard exposure within the urban healthcare system under tsunami conditions. Healthcare system vulnerability cannot be understood solely by the physical location of healthcare institutions. Rather, both (1) direct physical exposure of institutions located within estimated tsunami inundation zones and (2) indirect functional exposure resulting from population redistribution toward non-inundated institutions must be considered. By examining tsunami hazard zones, population distribution, and healthcare infrastructure across administrative boundaries, this study provides an integrated perspective that has been relatively underexplored.

Direct physical exposure of healthcare institutions

Several hospitals in Nishinari Ward were located within estimated tsunami inundation zones, indicating a substantial risk of structural damage or functional disruption during tsunami events. In such scenarios, these institutions may lose their capacity to provide medical services precisely when demand increases.

Previous disaster medicine research has reported cases in which hospitals themselves were damaged and experienced suspension or severe limitations of services [16]. This reflects a structural challenge in disaster settings, where demand increases while supply simultaneously declines. From this perspective, the location of healthcare institutions within hazard zones represents a critical component of system vulnerability.

Although prior GIS-based studies have evaluated tsunami risk and evacuation accessibility [17], relatively few have examined the hazard exposure of healthcare institutions themselves in metropolitan contexts. By focusing on healthcare infrastructure, this study contributes to a more comprehensive understanding of urban disaster vulnerability.

Indirect functional exposure associated with population redistribution

In addition to direct physical exposure, this study highlights indirect functional exposure resulting from population redistribution. When institutions located within inundation zones were assumed to be non-functional, the surrounding population of non-inundated institutions increased substantially, in some cases reaching approximately 5.5 times baseline levels. This suggests that institutions not directly affected by inundation may nevertheless experience substantial strain due to concentrated demand. Evidence from the Great East Japan Earthquake shows that medical demand increased significantly in affected areas, with many patients seeking care at nearby functioning facilities and temporary medical stations [18]. Similarly, previous GIS-based analyses in Osaka City have suggested that large-scale disaster scenarios may lead to shortages in hospital bed capacity [19].

These findings should also be interpreted in light of underlying socioeconomic and health-related differences between the study areas. Prior research has shown that socioeconomic conditions are associated with disparities in health outcomes, including higher tuberculosis incidence in certain districts of Osaka City such as Nishinari Ward [20-22]. These contextual factors may increase healthcare needs and create access barriers during disasters.

Taken together, the observed concentration of healthcare demand reflects not only spatial hazard exposure but also its interaction with pre-existing social and health-related vulnerabilities. This interaction may increase functional stress on healthcare systems, particularly in densely populated urban environments. Disaster preparedness should therefore incorporate both spatial and social dimensions.

Cross-boundary implications in metropolitan areas

Healthcare demand and evacuation behavior may extend beyond administrative boundaries. The spatial distribution of population and evacuation-related facilities suggests that residents may seek services across neighboring wards during disasters.

Previous studies have shown that cross-boundary coordination can reduce disaster-related risks [23,24]. In large metropolitan areas, where population density and service interdependence are high, such dynamics are likely to be particularly important.

These findings indicate that disaster preparedness planning should not be confined to administrative units. A broader regional perspective that incorporates population redistribution and inter-area coordination of healthcare resources is required. Planning frameworks based solely on administrative boundaries may therefore be insufficient, underscoring the need for regional medical coordination systems. The use of mesh-level data allows for fine-grained spatial analysis, while ward-level interpretation provides contextual understanding without altering the analytical scale.

The findings of this study suggest the potential for increased system stress under tsunami conditions; however, this should be interpreted as a hypothesized implication rather than a directly demonstrated outcome. This is because the analysis does not incorporate operational healthcare capacity, system performance metrics, or real-world behavioral responses. Therefore, the results should be understood as indicative of possible system vulnerability rather than definitive evidence of system failure.

Socioeconomic vulnerability is discussed in this study as contextual information to aid interpretation of spatial patterns; however, it was not incorporated into the spatial analysis. Therefore, references to socioeconomic factors should be interpreted as contextual considerations rather than analytical findings derived from the data.

The assumption that healthcare institutions within inundation zones become non-functional represents a simplified and conservative scenario. This approach is consistent with disaster preparedness frameworks that emphasize worst-case conditions in the absence of detailed facility-level resilience data. However, in real-world settings, partial functionality or variability in damage may occur. Therefore, the results should be interpreted as indicative of potential system stress under severe conditions rather than precise estimates.

This study presents deterministic estimates based on spatial allocation and does not incorporate statistical measures of uncertainty or variability (e.g., range or interquartile range). The results should therefore be interpreted as indicative spatial patterns rather than statistically derived distributions. In addition, sensitivity analyses (e.g., alternative allocation rules or small spatial perturbations) were not conducted. The potential impact of these assumptions is acknowledged as a limitation.

Methodological considerations and limitations

Several limitations should be noted. First, this study used spatial analysis of secondary data and did not assess operational capacities of healthcare institutions, such as bed availability, staffing, or disaster response capability. Therefore, actual surge capacity remains uncertain.

Second, population redistribution was estimated using a simplified nearest-medical-institution allocation model. In real disaster situations, transportation conditions, infrastructure damage, individual behavior, and inter-hospital coordination may influence patient flows. The use of Euclidean distance and a nearest-neighbor allocation approach may not fully reflect real-world accessibility patterns, including transportation networks and travel constraints.

This study does not account for differences in medical specialization or service capacity among healthcare institutions, which may influence actual healthcare utilization patterns under disaster conditions. In addition, sensitivity scenarios (e.g., partial functionality of healthcare facilities) were not incorporated, and the assumption of complete non-functionality may lead to overestimation of demand redistribution. Furthermore, uncertainty and robustness of the results to alternative modeling assumptions were not evaluated, which may influence the magnitude and spatial distribution of estimated demand.

In addition, this study also does not incorporate road network structures or travel-time constraints in the spatial allocation process. In addition, factors such as congestion, infrastructure disruption, and behavioral responses (e.g., hospital choice, avoidance, or triage routing) are not considered. Therefore, the results should be interpreted as simplified representations of potential spatial patterns rather than realistic simulations of healthcare access under disaster conditions. Future studies incorporating network-based accessibility and behavioral factors would provide more realistic estimates of healthcare demand and system performance.

Third, the inundation zones were based on hazard map estimates, and actual impacts may vary depending on event-specific conditions.

Finally, this study focused on two wards in Osaka City; therefore, generalizability to other settings should be interpreted with caution.

Implications for future research

Future studies should incorporate facility-level data, including bed capacity, staffing, and service structure, to better evaluate healthcare system response during disasters. In addition, travel-time and route-based analyses would enable more dynamic assessment of access.

Simulation models that integrate both functional loss of exposed institutions and demand concentration on non-exposed institutions would provide a more comprehensive evaluation of system resilience. Developing integrated frameworks that combine spatial analysis, demand estimation, and healthcare resource data would be important for advancing disaster preparedness in urban settings.

## Conclusions

Overall, this study suggests that disaster vulnerability in urban healthcare systems can be characterized by a dual structure consisting of direct physical exposure and indirect functional exposure. In large coastal metropolitan areas, effective disaster preparedness should consider not only static hazard exposure but also dynamic processes such as population redistribution and cross-boundary healthcare demand. Integrating these perspectives is important for developing more resilient and adaptive healthcare planning strategies.
